# Clinical-pharmacogenetic models for personalized cancer treatment: application to malignant mesothelioma

**DOI:** 10.1038/srep46537

**Published:** 2017-04-19

**Authors:** Katja Goričar, Viljem Kovač, Vita Dolžan

**Affiliations:** 1 Pharmacogenetics Laboratory, Institute of Biochemistry, Faculty of Medicine, University of Ljubljana, Ljubljana, Slovenia; 2 Institute of Oncology Ljubljana, Ljubljana, Slovenia

## Abstract

Large interindividual differences in treatment outcome are observed in cancer patients undergoing chemotherapy. Our aim was to develop and validate clinical-pharmacogenetic prediction models of gemcitabine/cisplatin or pemetrexed/cisplatin treatment outcome and develop an algorithm for genotype-based treatment recommendations in malignant mesothelioma (MM). We genotyped 189 MM patients for polymorphisms in gemcitabine, pemetrexed and cisplatin metabolism, transport and drug target genes and DNA repair pathways. To build respective clinical-pharmacogenetic models, pharmacogenetic scores were assigned by rounding regression coefficients. Gemcitabine/cisplatin model was based on training group of 71 patients and included CRP, histological type, performance status, *RRM1* rs1042927, *ERCC2* rs13181, *ERCC1* rs3212986, and *XRCC1* rs25487. Patients with higher score had shorter progression-free (PFS) and overall survival (*P* < 0.001). This model’s sensitivity was 0.615 and specificity 0.812. In independent validation group of 66 patients the sensitivity and specificity were 0.667 and 0.641, respectively. Pemetrexed/cisplatin model was based on 57 patients and included CRP, *MTHFD1* rs2236225, and *ABCC2* rs2273697. Patients with higher score had worse response and shorter PFS (*P* < 0.001). This model’s sensitivity was 0.750 and specificity 0.607. In independent validation group of 20 patients the sensitivity and specificity were 0.889 and 0.500, respectively. The proposed algorithm based on these models could enable the choice of the most effective chemotherapy for 85.5% of patients and lead to improved treatment outcome in MM.

Research in the field of personalized medicine focuses on biomarkers that could help customize therapy for individual patients, thus leading to more effective treatment with fewer adverse events. Especially in oncology, several tumor markers have been identified and it has been shown for example in lung cancer that personalized treatment approach could improve treatment outcome, therefore patient stratification based on tumor mutations is already required before targeted treatment^1^. It has been suggested that apart from somatic mutations, interindividual variability in genes coding for drug metabolizing enzymes, drug transporters, drug targets or proteins involved in DNA repair could be used as a blood biomarker for guiding treatment selection^2^. Personalized treatment approach based on genetic biomarkers could therefore improve the outcome of cancer treatment.

Malignant mesothelioma (MM) is an aggressive malignancy with poor prognosis, usually associated with exposure to asbestos^3^. Introduction of chemotherapy significantly improved survival of MM patients; in Slovenia median overall survival increased from 5.6 to 14.5 months^3^,^4^. A randomized clinical trial has shown that treatment with pemetrexed/cisplatin combination improved outcome in MM patients, therefore, it became the standard treatment^5^. Comparable results were obtained for gemcitabine/cisplatin doublet^3^,^6^,^7^,^8^.

Despite improved survival, response rates to chemotherapy in MM are still only up to 40%^3^,^9^, and biomarkers that could improve response rate are needed. So far, no target mutations were identified in MM that could guide targeted treatment. MM treatment outcome was associated with clinical characteristics^10^ and genetic variability in drug transport, metabolism and target genes and DNA repair pathways^11^,^12^,^13^,^14^.

Gemcitabine is a nucleoside analog that inhibits ribonucleotide reductase M1 (RRM1) and decreases deoxyribonucleotide pools for DNA synthesis, while its incorporation into DNA leads to accumulation of strand breaks^15^. In our previous studies, single nucleotide polymorphisms (SNPs) in the target *RRM1* gene, and in DNA repair genes *ERCC1, ERCC2*, and *XRCC1* were associated with survival in MM patients^11^,^12^,^13^.

Pemetrexed is a folic acid analogue that inhibits several key folate pathway enzymes, leading to impaired DNA synthesis^16^. In our previous study, response to pemetrexed was associated with SNPs in folate pathway gene *MTHFD1* and efflux transporter gene *ABCC2*^14^.

Although we have previously identified pharmacogenetic markers that may influence MM treatment outcome, these results have not been used yet for treatment guidance^11^,^12^,^13^,^14^. Translation of pharmacogenetic results into clinical practice can be challenging, especially as they are seldom incorporated in actionable forms such as scores or dosing guidelines. Pharmacogenetic models including both clinical and genetic parameters could be a useful tool that would help guide treatment selection^17^,^18^. In addition to pharmacogenetic models, algorithms that would facilitate translation into clinical practice are needed.

Our aim was to construct clinical-pharmacogenetic models for gemcitabine/cisplatin or pemetrexed/cisplatin treatment outcome and to develop an algorithm for genotype-based treatment recommendations that could facilitate individualization of MM treatment.

## Materials and Methods

### Patients

All patients with histologically proven MM, that started treatment with gemcitabine/cisplatin or pemetrexed/cisplatin based chemotherapy at the Institute of Oncology Ljubljana, Slovenia between March 2002 and September 2013, as well as patients that started with pemetrexed/cisplatin treatment between 2014 and 2016, were included in the study.

Most of the patients were diagnosed at the University Clinic of Pulmonary and Allergic Diseases in Golnik, Slovenia. Patient data were obtained from medical records or assessed during clinical interview. Written informed consent was obtained for all patients. The study was approved by the Slovenian Ethics Committee for Research in Medicine and was carried out according to the Declaration of Helsinki.

### Survival and response assessment

Primary endpoint evaluated in the study was progression-free survival (PFS), defined as time from the beginning of first or second line chemotherapy with a particular drug to the progression or death of any cause. Tumor response was evaluated using modified Response Evaluation Criteria in Solid Tumors (RECIST)^19^. Response rate was defined as percentage of patients achieving partial or complete response. Overall survival (OS) was defined as time from the beginning of first or second line chemotherapy with a particular drug to death of any cause. Patients without progression or death at the time of the analysis were censored at the date of the last follow-up.

### DNA extraction and genotyping

EDTA-stabilized blood samples for DNA extraction were collected at the time of diagnosis. Extraction of genomic DNA was performed using Qiagen FlexiGene kit (Qiagen, Hilden, Germany) according to the manufacturer’s instructions.

The selection of genetic and clinical variables was based on results of our previous studies where pathway-based approach was used to identify factors influencing tumor response, PFS or OS in Slovenian MM patients^11^,^12^,^13^,^14^. In this study genotyping for all polymorphisms in gemcitabine, pemetrexed and cisplatin metabolism, transport and drug target genes and DNA repair pathways significantly associated with treatment outcome in previous studies was extended to all patients. *MTHFD1* rs2236225 (p.Arg653Gln) and *XRCC1* rs25487 (p.Arg399Gln) polymorphisms were determined using TaqMan SNP Genotyping assays according to the manufacturer’s instructions (Applied Biosystems, Foster City, CA). *ABCC2* rs2273697 (p.Val417Ile), *RRM1* rs1042927 (3′ untranslated region), *ERCC2* rs13181 (p.Lys751Gln), and *ERCC1* rs3212986 (3′ untranslated region) polymorphisms were genotyped using KASPar assays according to the manufacturer’s instructions (KBiosciences, Herts, UK). Genotyping was performed blinded regarding the study endpoints and repeated in 20% samples to check for genotyping accuracy.

### Model building and statistical analyses

For building a gemcitabine/cisplatin clinical-pharmacogenetic model, 71 patients with complete genetic information on the investigated pharmacogenes available from our previously published studies were included in the training group^11^,^12^,^13^. The gemcitabine/cisplatin validation group consisted of 66 patients from previously published studies that required additional genotyping as well as patients diagnosed and treated after the completion of previous studies. Because pemetrexed/cisplatin is not frequently used in Slovenia, all 57 patients treated with this combination as first or second line regimen between 2002 and 2013 were included in the training group used for building a clinical-pharmacogenetic model. More specifically, we included patients participating in a randomized clinical trial (Trial registration ID: NCT01281800) from our previous study^14^, and more recently included patients from the trial as well as patients treated outside the clinical trial. The pemetrexed/cisplatin validation group consisted of twenty patients starting treatment after September 2013.

Pharmacogenetic scores for the clinical-pharmacogenetic model were assigned by rounding the absolute values of regression coefficients from PFS analysis. For each patient, the combined pharmacogenetic score was calculated by summing up all individual scores of variables included in the model. Higher scores indicated shorter survival. To evaluate the discriminative performance of the model, we compared how many patients had PFS above or below the median in each group. A receiver operating characteristic (ROC) curve was derived to evaluate the discriminative performance of the model and area under the curve (AUC) was determined. Specificity, sensitivity, positive predictive value (PPV) and negative predictive value (NPV) were calculated. In survival analysis, Cox proportional hazards model was used and hazard ratio (HR) with 95% confidence interval (CI) was determined. All statistical tests were two-sided and the level of significance was set to 0.05. All statistical analyses were carried out using IBM SPSS Statistics, version 19.0 (IBM Corporation, Armonk, NY, USA).

## Results

### Patients’ characteristics

We included 189 MM patients. The training and validation groups of gemcitabine/cisplatin treated patients included 71 and 66 patients, respectively. Among gemcitabine/cisplatin treated patients, those in the validation group tended to be older (*P* = 0.050) and had significantly worse performance status (*P* = 0.005) than patients in the training group. In the validation group, patients also has higher CRP, but the difference was not statistically significant (*P* = 0.084). In the training group, 11 (15.5%) patients received surgical treatment and had significantly longer PFS (*P* = 0.006; HR = 0.35; 95% CI = 0.16–0.74), but not OS (*P* = 0.123; HR = 0.54; 95% CI = 0.24–1.18). However, eligibility for surgical treatment significantly correlated with low CRP (*P* = 0.015) and epithelioid histological type (*P* = 0.021), therefore it did not contribute to the clinical-pharmacogenetic model. Significantly less patients in the validation group received surgical treatment (*P* = 0.004).

The training and validation groups of pemetrexed/cisplatin treated patients included 57 and 20 patients, respectively. In the training group, 32 (56.1%) patients received pemetrexed as first line treatment (Table 1). There were no statistically significant differences between training and validation group regarding their demographic and clinical characteristics (Table 1). In the training group, only 3 (5.3%) patients received surgical treatment, and there was no significant association with PFS (*P* = 0.105; HR = 0.19; 95% CI = 0.03–1.42) or OS (*P* = 0.349; HR = 0.39; 95% CI = 0.05–2.84), but it again correlated with low CRP (*P* = 0.016).

### Gemcitabine/cisplatin clinical-pharmacogenetic model

In the training group longer survival was associated with CRP below 23 mg/l, better performance status or non-sarcomatoid histology and four of the investigated polymorphisms. Among them *RRM1* rs1042927 AA, *ERCC2* rs13181 AA, *ERCC1* rs3212986 CC, and *XRCC1* rs25487 CC genotypes were associated with longer PFS and OS. The final clinical-pharmacogenetic model had scores between 0 and 3.4, with higher scores indicating shorter survival (Table 2).

**Figure 1:**
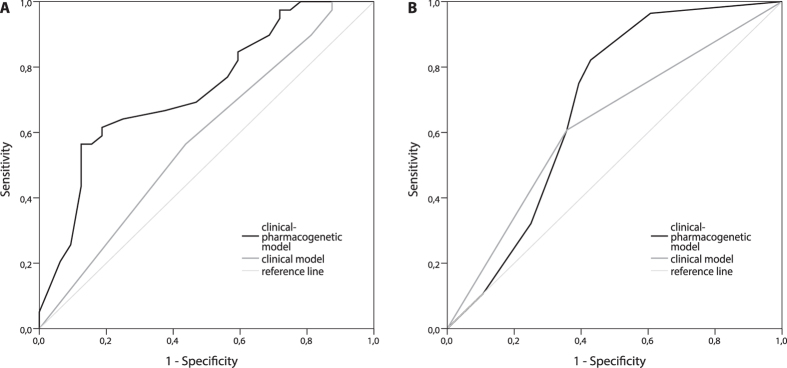
Receiver operating characteristic curve for predicting gemcitabine (**A**) and pemetrexed (**B**) treatment outcome in the training group for clinical-pharmacogenetic model and clinical parameters.

**Figure 2:**
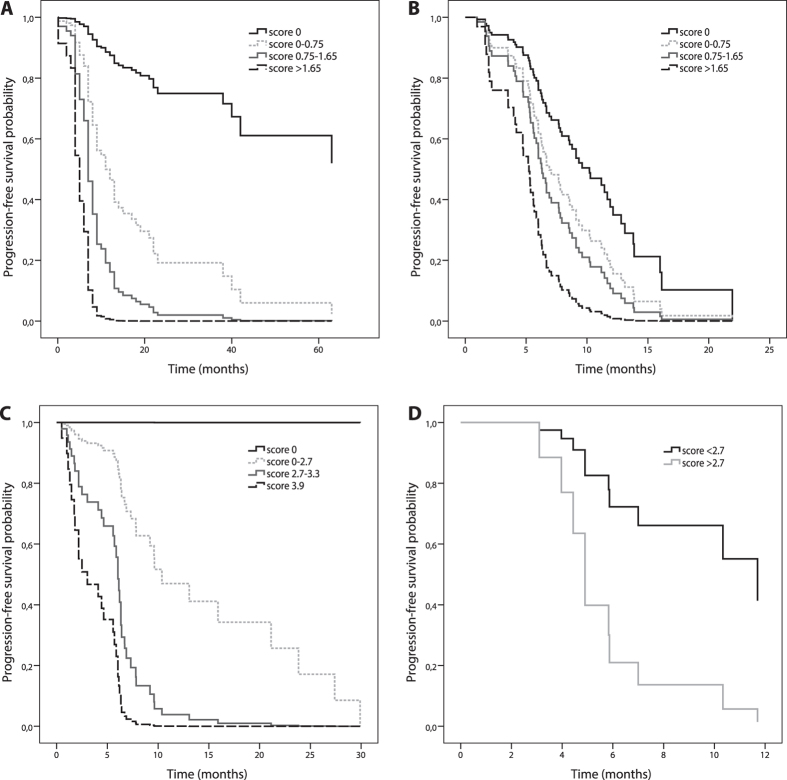
Differences in progression-free survival of MM patients treated with gemcitabine based on clinical-pharmacogenetic model in the training (**A**) and validation (**B**) cohort. Differences in progression-free survival of MM patients treated with pemetrexed based on clinical-pharmacogenetic model in the training (**C**) and validation (**D**) cohort.

This clinical-pharmacogenetic model had better predictive value compared to the clinical model that included only clinical characteristics. The AUC for predicting PFS above or below 8 months was 0.732 (95% CI = 0.614–0.849; *P* = 0.001) for the clinical-pharmacogenetic model and 0.581 (95% CI = 0.446–0.716, *P* = 0.243) for the clinical model (Fig. 1A). In the clinical-pharmacogenetic model, cutoff score of 0.75 had sensitivity of 0.615 and specificity of 0.812, with PPV 0.800 and NPV 0.634 (Table 3). Patients with scores above cutoff had significantly shorter PFS (*P* < 0.001; HR = 2.75; 95% CI = 1.75–4.32; Fig. 2A) and OS (*P* < 0.001; HR = 2.77; 95% CI = 1.73–4.44) compared to patients with lower scores. Median PFS and OS for patients with scores below 0.75 were 13.0 (8.0–24.5) and 26.0 (16.0–41.3) months, respectively, compared to 7.0 (5.0–9.0) and 14.0 (9.0–19.5) months for patients with scores above 0.75.

In the validation group, PPV of the clinical-pharmacogenetic model for PFS above or below 6 months was 0.735, while NPV was 0.563 (Table 3). Sensitivity was 0.667 and specificity was 0.641. Patients with scores above the cutoff value of 0.75 had significantly shorter PFS (*P* = 0.020; HR = 1.52; 95% CI = 1.07–2.17; Fig. 2B) and OS (*P* = 0.036; HR = 1.57; 95% CI = 1.03–2.38) compared to patients with lower scores. For example, median PFS and OS were 11.7 (3.5–11.7) and 12.8 (6.9–12.8) months in patients who scored 0, but only 3 (1.9–6.9) and 4.7 (2.1–10.3) months in patients who scored above 1.65.

### Pemetrexed/cisplatin clinical-pharmacogenetic model

In this model CRP below 23 mg/l, *MTHFD1* rs2236225 GG genotype and *ABCC2* rs2273697 GA + AA genotypes were associated with better response rate or PFS. The final clinical-pharmacogenetic model had values ranging between 0 and 3.9 (Table 2).

Also for this treatment regimen, the clinical-pharmacogenetic model improved predictive value as compared to the clinical model that included only CRP. AUC for predicting PFS longer or shorter than 6 months was 0.684 (95% CI = 0.538–0.829; *P* = 0.018) for the clinical-pharmacogenetic model and 0.625 (0.477–0.773, *P* = 0.108) for CRP (Fig. 1B). Cutoff score of 2.7 had sensitivity of 0.750 and specificity of 0.607, PPV was 0.656 and NPV was 0.708. Patients with higher scores had significantly shorter PFS (*P* < 0.001, HR = 2.73; 95% CI = 1.86–4.00, Fig. 2C) and OS (*P* < 0.001, HR = 2.45; 95% CI = 1.64–3.65). Median PFS and OS for patients with scores below 2.7 were 8.1 (4.5–15.9) and 11.3 (4.7–23.9) months, respectively, compared to 4.8 (1.5–6.2) and 7.6 (4.4–10.9) months for patients with scores above 2.7. Eleven out of twelve patients (91.7%) with maximal score of 3.9 had PFS below 6 months (Table 3).

Response rate was also worse in patients with higher scores. Eleven (78.6%) out of 14 patients that responded well to pemetrexed/cisplatin had scores below 2.7. NPV was 0.875, but PPV was only 0.367. This may be due to the fact that nearly half of patients received pemetrexed/cisplatin as second line treatment and among those only 12.0% responded well.

The model performed better in first line treatment, showing highly significant association with both PFS and OS (*P* < 0.001, HR = 2.82; 95% CI = 1.74–4.58 and *P* < 0.001, HR = 3.01; 95% CI = 1.71–5.29, respectively). Median PFS and OS for patients in the first line treatment with scores below 2.7 were 10.4 (6.4–15.9) and 15.8 (10.1–29.5) months, respectively, compared to 5.6 (1.8–6.8) and 10.0 (7.1–12.3) months for patients with scores above 2.7. PPV for prediction of response rate was 0.526, while NPV was 0.923. No patient with score 0 experienced disease progression, while all thirteen patients with score above 2.7 progressed.

Survival times were shorter in second line treatment, but the model remained significantly associated with PFS (*P* = 0.009, HR = 2.15; 95% CI = 1.21–3.82) and OS (*P* = 0.048, HR = 1.89; 95% CI = 1.01–3.56). No patient with score of 0 experienced disease progression, while 9 out of 11 (81.8%) patients with score above 2.7 progressed.

In the validation group, the clinical-pharmacogenetic model for PFS above or below 6 months reached PPV 0.615, while NPV was 0.833 (Table 3). Sensitivity of this model was 0.889 and specificity was 0.500. Patients with scores above the cutoff value of 2.7 had significantly shorter PFS (*P* = 0.030; HR = 4.81; 95% CI = 1.16–19.91; Fig. 2D). Median PFS was 4.2 (3.8–6.4) months in patients with scores above 2.7 and 6.6 (4.9–12.0) months in patients with scores below 2.7. Due to shorter follow-up and low number of events, we did not perform OS analysis in this group.

### Treatment stratification algorithm

**Figure 3:**
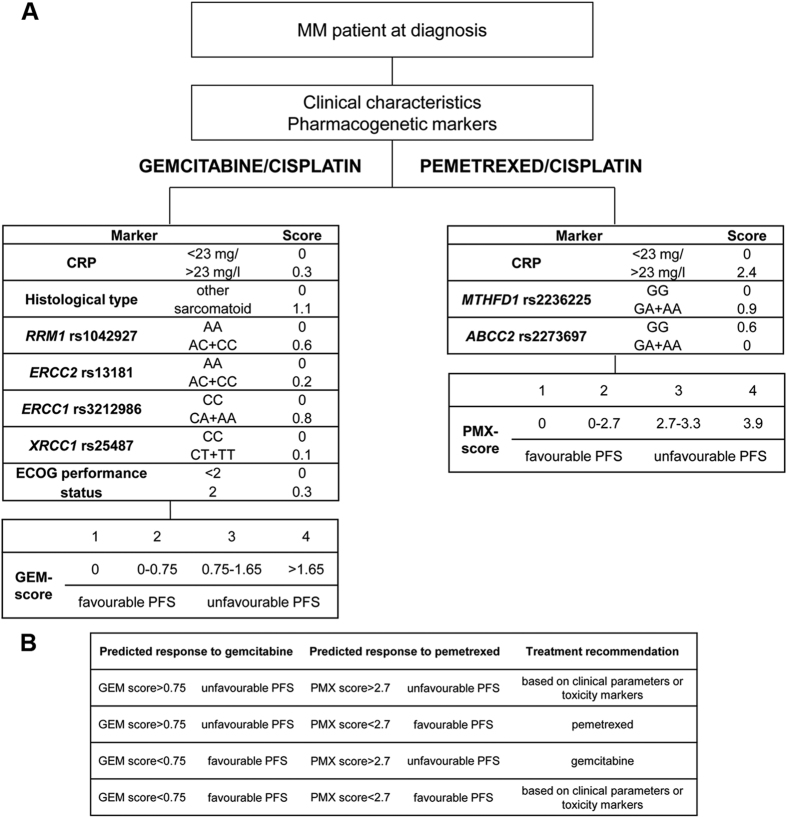
Algorithm for the prediction of outcome of gemcitabine/cisplatin or pemetrexed/cisplatin treatment based on the developed clinical-pharmacogenetic model (**A**) and algorithm-based treatment recommendations (**B**). CRP, C-reactive protein; ECOG, Eastern Cooperative Oncology Group; GEM, gemcitabine, PFS, progression-free survival; PMX, pemetrexed.

Based on the developed and validated clinical-pharmacogenetic models we proposed an algorithm for stratification of patients into distinct treatment groups (Fig. 3). Genotyping data for SNPs included in both models were available for 159 patients. Based on the algorithm, a more favorable chemotherapy regimen could be recommended in 64.2% of patients: gemcitabin/cisplatin in 28.3% and pemetrexed/cisplatin in 35.9%. The algorithm predicted that 21.4% of patients would respond equally well to both treatments, but 14.5% of patients would probably not respond well to either. Based on our algorithm altogether 85.5% of patients could be treated using the most effective of the two chemotherapeutic regimens.

## Discussion

Based on the developed clinical-pharmacogenetic models for pemetrexed/cisplatin or gemcitabine/cisplatin chemotherapy outcomes we have proposed an algorithm that allows genotype-based treatment recommendations in MM patients.

Our gemcitabine/cisplatin clinical-pharmacogenetic model included CRP level, histological type, performance status and four SNPs: *RRM1* rs1042927, *ERCC2* rs13181, *ERCC1* rs3212986, and *XRCC1* rs25487. The model was developed based on results of our previous studies^11^,^12^,^13^ as we were not aware of any studies investigating the influence of SNPs on outcome of gemcitabine/cisplatin treatment in MM, while *ERCC1, RRM1, ERCC2* and *XRCC1* were consistently identified as prediction factors in pancreatic or non-small cell lung cancer (NSCLC)^20^,^21^. Patients with higher scores had significantly shorter PFS and OS. Patients in the validation group were older, had worse performance status and higher CRP and were generally not amenable to surgery and consequently also had worse treatment outcome. As patients were enrolled continuously, this difference occurred by chance and was not related to patient selection. Although this difference represents a limitation of our study, we confirmed the predictive value of the model with PPV of 0.800 in the training and 0.735 in the validation group even in patients with shorter survival.

Our pemetrexed/cisplatin clinical-pharmacogenetic model included two SNPs, *MTHFD1* rs2236225 and *ABCC2* rs2273697, and CRP level. Other clinical characteristics included in the gemcitabine/cisplatin model were not as important in pemetrexed-treated patients. Patients with higher scores in the final model had significantly shorter PFS and OS, as well as worse response rate, especially if patients had all the markers associated with worse outcome. The effect was even more prominent in patients receiving pemetrexed/cisplatin in the first line of chemotherapy. Patients treated in the second line of chemotherapy had worse response and shorter survival in general, which was a limitation of this study. However, even in these patients the model was still significantly associated with both PFS and OS. As pemetrexed/cisplatin combination started to be used only recently for treatment of MM in Slovenia, only twenty patients with shorter follow-up time could be included the validation group. The clinical-pharmacogenetic model remained significantly associated with PFS with PPV of 0.615 and NPV of 0.833. Higher number of false positives – patients with low scores but shorter PFS in this group could in part be due to shorter follow-up period in these patients. Therefore our results should be considered preliminary and further validation is necessary. Nevertheless, our model was valid even in patients with poor performance status and in second line treatment.

Another limitation of our study is the large contribution of CRP to the model, as increased CRP is required for unfavorable prognosis prediction. Therefore, inclusion of other genetic factors or molecular markers could help refine the current model. Most studies evaluating response to pemetrexed in MM focused on tumor markers. TYMS mRNA and protein expression levels in tumor were associated with response but similar to our results *TYMS* promoter polymorphisms did not play a role^14^,^22^,^23^. A deletion in *TYMS* 3′ untranslated region has been associated with PMX treatment outcome in MM^23^, but studies in NSCLC gave inconsistent results, suggesting further studies are needed regarding the role of this polymorphism^24^,^25^.

Despite advances in cancer treatment, selection of the optimal treatment regimen for MM patients remains challenging, especially due to a lack of appropriate randomized clinical trials showing survival benefit. However, population-based studies on unselected populations have shown that overall survival increased significantly on national levels with the use of chemotherapy in Slovenia, the Netherlands, Norway and USA^3^,^4^,^9^,^26^. Even though other factors such as improved diagnostics and best supportive care could partly contribute to this improvement, these results suggest chemotherapy is beneficial for most MM patients. Different chemotherapy regimens are available for MM treatment, and several studies were performed to compare them. Among the randomized trials, Vogelzang *et al*. were the first to demonstrate that pemetrexed/cisplatin doublet chemotherapy is more effective in MM than cisplatin monotherapy^5^. A few other combinations were evaluated in randomized trials, but they did not show an important improvement of OS (reviewed in ref. 27), while several phase II or phase III clinical trials have shown some combinations are comparable to pemetrexed/cisplatin doublet regarding treatment outcome (reviewed in ref. 3). Studies consistently show that treatment with gemcitabine/cisplatin doublet achieves comparable results^6^,^7^,^8^,^28^. In Slovenian randomized phase II trial comparing pemetrexed/cisplatin and gemcitabine/cisplatin, overall median OS reached 18.6 months and there were no significant differences between groups treatment^29^. Still, more randomized trials are needed to further improve MM treatment, both first line and second line. Additionally, treatment guidelines should include biomarkers that could help guide treatment selection.

Our most important contribution is the proposed simple algorithm for treatment selection based on both gemcitabine/cisplatin and pemetrexed/cisplatin models that could be used for translation into clinical practice. Patients’ clinical characteristics and genotypes of six SNPs allowed prediction of PFS after gemcitabine/cisplatin or pemetrexed/cisplatin treatment. Our model predicted that if all the patients included in our study were treated with the standard pemetrexed/cisplatin chemotherapy, this therapy would be effective in 57.3% of patients. However, using the proposed algorithm, 85.5% of patients could be stratified into an effective chemotherapy regimen. In 14.5% of MM patients with predicted poor response to both chemotherapy regimens treatment could be guided based on clinical parameters as so far or based on pharmacogenetic markers of toxicity identified in our previous studies^11^,^12^,^13^,^14^. On the other hand such early information about the high risk of poor outcome of the standard treatment could be important to spare these patients the non-effective treatment with frequent adverse events decreasing the quality of life and to direct them into clinical trials of new treatment approaches. For example, these patients might benefit from new immunotherapy approaches that target immune checkpoints or mesothelin^30^,^31^. As MM is a polyclonal malignancy, there are no specific driver mutations involved in disease development or progression^32^. On the other hand, recent next-generation sequencing studies have shown that despite low number of mutations in individual tumors, mutations were more often present in some pathways such as p53/DNA repair pathway, cell cycle, or phosphatidylinositol 3-kinase-AKT pathway, or genes coding for epigenetic modifiers^32^,^33^. Additionally, copy number variations, especially minute deletions, were commonly present in these pathways^34^, suggesting they are often impaired in MM and could potentially be targeted in novel treatment approaches developed for MM. Several approaches are currently being investigated as an option for improving MM treatment, but further studies are needed to select optimal combination therapies^30^,^31^, likely incorporating novel concepts such as network analysis or even drug repurposing^35^.

Although our results are promising, some issues apart from validation in an independent population should be addressed before implementation in the clinic, mainly the most appropriate methodology or availability of approved tests and cost-effectiveness of genotyping. The additional cost of genotyping was small compared to high costs of chemotherapy itself. Our model can be further improved by including additional clinical factors and biomarkers. Other clinical factors such as pain were also associated with disease prognosis previously^10^. Tumor stage is often associated with treatment outcome, however we did not include it in our model due to significant correlations with other clinical parameters and because stage is not determined in peritoneal mesothelioma. Some patients in our study were amenable to surgery and this was associated with longer survival, consistently with other studies^23^,^26^. However, surgical treatment was not an independent prognostic factor in our study, but rather a factor associated with CRP, histological type and generally favorable performance status. The role of surgical treatment, especially extra-pleural pneumonectomy in MM is still debated^36^,^37^,^38^. Studies suggest selected population of MM patients benefits from surgery as part of multi-modal treatment, but further studies are needed to determine the optimal type of surgery and which patients should receive it^37^. Moreover, other genetic factors have been associated with differences in gemcitabine/cisplatin or pemetrexed/cisplatin treatment outcome in other malignancies and inclusion of more SNPs could perhaps improve the prediction of clinical-pharmacogenetic models^23^,^39^,^40^,^41^. Tumor mRNA expression of some DNA repair enzymes could also serve as an additional prognostic marker^42^.

The advantage of DNA-based or other blood-based markers is that they do not require cyto- or histopathological material, are non-invasive, and their determination can be easier and faster compared to histopathological tumor markers. Importantly, SNP genotyping is also reliable and less expensive. Clinical-pharmacogenetic models combining different clinical and pharmacogenetics markers have several advantages over single markers, which are unlikely to be able to explain all the variability in response. Moreover, SNP-based clinical-pharmacogenetic models could be easily introduced into the clinic to help guide treatment selection: easily calculated scores would enable fast classification of patients into one of the recommended treatment groups and consequently a more personalized treatment approach.

Treatment stratification based on tumor markers is currently mostly used in treatment with targeted drugs. However, even in advanced NSCLC, where various biomarkers have already been described and the analysis of *EGFR* and *ALK* mutations is clinically available, most patients are stratified for chemotherapy based on histological data only^1^,^20^,^21^,^39^,^43^,^44^,^45^,^46^,^47^. Studies have shown that patient stratification based on RRM1 and ERCC1 expression or SNPs could improve outcome in NSCLC, but these approaches are not routinely used yet^44^,^45^. As gemcitabine/cisplatin or pemetrexed/cisplatin chemotherapy doublets are also used in advanced NSCLC, our SNP-based clinical-pharmacogenetic models could be extended to this treatment, if confirmed in this group of patients.

In conclusion, we have developed clinical-pharmacogenetic models for predicting gemcitabine/cisplatin and pemetrexed/cisplatin treatment outcome. Using the proposed algorithm, effective chemotherapy could be recommended for 85.5% of MM patients, however this needs to be confirmed in a prospective study. Similar approach could be used for selecting the most favorable treatment option and thus improving outcomes of chemotherapy in other cancers where more treatment options are available.

## Additional Information

**How to cite this article:** Goričar, K. *et al*. Clinical-pharmacogenetic models for personalized cancer treatment: application to malignant mesothelioma. *Sci. Rep.* **7**, 46537; doi: 10.1038/srep46537 (2017).

**Publisher's note:** Springer Nature remains neutral with regard to jurisdictional claims in published maps and institutional affiliations.

## Figures and Tables

**Table 1:** Characteristics of malignant mesothelioma patients receiving gemcitabine/cisplatin or pemetrexed/cisplatin chemotherapy.

Characteristic		Treatment with gemcitabine			Treatment with pemetrexed		
		Training group (*N* = 71); *N* (%)	Validation group (*N* = 66); *N* (%)	P^a^	Training group (*N* = 57); *N* (%)	Validation group (*N* = 20); *N* (%)	P^a^
Gender	Male	52 (73.2)	52 (78.8)	0.448^b^	46 (80.7)	13 (65.0)	0.153^b^
	Female	19 (26.8)	14 (21.2)		11 (19.3)	7 (35.0)	
Age	Median (25–75%)	61 (54–69)	65.5 (57.5–71.3)	0.050^c^	63 (59–69)	62.5 (56–70)	0.653^c^
Stage	I	6 (8.5)	3 (4.5)		4 (7.0)	1 (5.0)	
	II	18 (25.4)	15 (22.7)		17 (29.8)	5 (25.0)	
	III	20 (28.2)	21 (31.8)	0.826^b^	18 (31.6)	9 (45.0)	0.839^b^
	IV	21 (29.6)	19 (28.8)		16 (28.1)	4 (20.0)	
	Peritoneal	6 (8.5)	8 (12.1)		2 (3.5)	1 (5.0)	
Histological type	Epitheloid	49 (69.0)	50 (75.8)		45 (78.9)	16 (80.0)	
	Biphasic	15 (21.1)	7 (10.6)		6 (10.5)	1 (5.0)	
	Sarcomatoid	5 (7.0)	7 (10.6)	0.380^b^	3 (5.3)	3 (15.0)	0.340^b^
	Not characterized	2 (2.8)	2 (3.0)		3 (5.3)	0 (0.0)	
ECOG performance status	0	38 (53.5)	20 (30.3)		26 (45.6)	5 (25.0)	
	1	25 (35.2)	26 (39.4)	0.005^b^	23 (40.4)	13 (65.0)	0.159^b^
	2	8 (11.3)	20 (30.3)		8 (14.0)	2 (10.0)	
Line of treatment	First line	71 (100)	66 (100)		32 (56.1)	20 (100)	
	Second line				25 (43.9)		
C-reactive protein	Median (25–75%)	21 (5–65)	29 (12.3–101)	0.084^c^	22 (6–62)	18.5 (11.5–36.8)	0.728^c^
Surgical treatment	No	60 (84.5)	65 (98.5)		54 (94.7)	19 (95.0)	
	Yes	11 (15.5)	1 (1.5)		3 (5.3)	1 (5.0)	
Response rate	CR or PR	40 (56.3)	18 (28.1)		14 (25.5)	10 (55.6)	
	SD or progress	27 (38.0)^d^	46 (71.9)^e^		41 (74.5)^e^	8 (44.4)	
Overall survival	Median (25–75%)	16 (10–28)	10.5 (6.2–15.1)		9.4 (4.7–16.2)^f^	8.2 (4.5–12.7)	
Progression-free survival	Median (25–75%)	8 (6–13)	6.4 (4.8–9.5)		6.1 (2.9–9.6)^f^	5.8 (4.5–11.3)	
Follow-up	Mean (95% CI)	62.3 (49.4–75.2)	21.1 (18.0–24.3)		19.8 (15.8–23.9)	11.3 (8.0–14.6)	

^a^comparison of clinical and demographic characteristics at diagnosis. ^b^calculated using chi square test. ^c^calculated using Mann-Whitney test. ^d^data missing for four patients. ^e^data missing for two patients. ^f^calculated from the beginning of pemetrexed-based chemotherapy. CI, confidence interval; CR, complete response; ECOG, Eastern Cooperative Oncology Group; N, number of patients; PR, partial response; SD, stable disease.

**Table 2:** Pharmacogenetic scores for the variables included in clinical-pharmacogenetic model predicting outcome of gemcitabine/cisplatin or pemetrexed/cisplatin chemotherapy.

Chemotherapy	Variable	HR	B	Score	
Gemcitabine	CRP	1.31	0.266	<23 mg/l	0
				>23 mg/l	0.3
	Histological type	2.97	1.088	Other	0
				Sarcomatoid	1.1
	*RRM1* rs1042927	1.85	0.615	AA	0
				AC + CC	0.6
	*ERCC2* rs13181	1.18	0.165	AA	0
				AC + CC	0.2
	*XRCC1* rs25487	1.06	0.054	CC	0
				CT + TT	0.1
	*ERCC1* rs3212986	2.33	0.846	CC	0
				CA + AA	0.8
	ECOG performance status	1.38	0.320	< 2	0
				2	0.3
Pemetrexed	CRP	11.25	2.420	< 23 mg/l	0
				> 23 mg/l	2.4
	*MTHFD1* rs2236225	2.37	0.864	GG	0
				GA + AA	0.9
	*ABCC2* rs2273697	0.54	−0.623	GG	0.6
				GA + AA	0

B, regression coefficient; CRP, C-reactive protein; ECOG, Eastern Cooperative Oncology Group; HR, hazard ratio.

**Table 3:** Comparison of observed and predicted treatment outcomes based on the gemcitabine clinical-pharmacogenetic model score (GEM-score) or the pemetrexed clinical-pharmacogenetic model score (PMX-score).

	Observed outcomes	Clinical-pharmacogenetic model score category *N* (%)				
		GEM-score 0	GEM-score 0–0.75	GEM-score 0.75–1.65	GEM-score >1.65	*P*
		Predicted PFS > median		Predicted PFS < median		
Gemcitabine training group	PFS > 8 months	2 (100.0)	22 (78.6)	15 (44.1)	0 (0.0)	< 0.001
	PFS < 8 months	0 (0.0)	6 (21.4)	19 (55.9)	7 (100.0)	
Gemcitabine validation group	PFS > 6 months	2 (66.7)	23 (74.2)	12 (50.0)	2 (25.0)	0.042
	PFS < 6 months	1 (33.3)	8 (25.8)	12 (50.0)	6 (75.0)	
	**Observed outcomes**	**PMX-score 0**	**PMX-score 0–2.7**	**PMX-score 2.7–3.3**	**PMX-score 3.9**	P
		**Predicted PFS > median**		**Predicted PFS < median**		
Pemetrexed training group	PFS > 6 months	3 (50.0)	18 (69.2)	6 (50.0)	1 (8.3)	0.004
	PFS < 6 months	3 (50.0)	8 (30.8)	6 (50.0)	11 (91.7)	
Pemetrexed validation group^a^	PFS > 6 months	0 (0.0)	8 (66.7)	1 (16.7)	/	0.099
	PFS < 6 months	1 (100.0)	6 (33.3)	5 (83.3)	/	

N, number of patients; PFS, progression-free survival.

^a^one patient censored before the earliest event was excluded from the analysis.
